# Experimental Models for the Study of Hereditary Cornification Defects

**DOI:** 10.3390/biomedicines9030238

**Published:** 2021-02-26

**Authors:** Dragan Copic, Maria Laggner, Polina Kalinina, Katharina Klas, Erwin Tschachler, Michael Mildner

**Affiliations:** 1 Laboratory for Cardiac and Thoracic Diagnosis, Regeneration and Applied Immunology, Department of Surgery, Research Laboratories, Medical University of Vienna, 1090 Vienna, Austria; dragan.copic@meduniwien.ac.at (D.C.); maria.laggner@meduniwien.ac.at (M.L.); katharina.klas@meduniwien.ac.at (K.K.); 2 Division of Thoracic Surgery, Medical University of Vienna, 1090 Vienna, Austria; 3 Department of Dermatology, Medical University of Vienna, 1090 Vienna, Austria; polina.kalinina@meduniwien.ac.at (P.K.); erwin.tschachler@meduniwien.ac.at (E.T.)

**Keywords:** ichthyosis, cornification defect, organotypic skin model, gene knockdown

## Abstract

Ichthyoses comprise a broad spectrum of keratinization disorders due to hereditary defects of cornification. Until now, mutations in more than 50 genes, mostly coding for structural proteins involved in epidermal barrier formation, have been identified as causes for different types of these keratinization disorders. However, due to the high heterogeneity and difficulties in the establishment of valid experimental models, research in this field remains challenging and translation of novel findings to clinical practice is difficult. In this review, we provide an overview of existing models to study hereditary cornification defects with focus on ichthyoses and palmoplantar keratodermas.

## 1. Introduction

The spectrum of hereditary disorders of cornification (DOC) consists of a variety of rare genodermatoses, such as ichthyoses and palmoplantar keratodermas [1,2]. For many of these diseases, the underlying pathological causes have been identified. Most frequently, these disorders develop due to monogenic alterations in genes important for the establishment or maintenance of a functional epidermal barrier. These genes are essential for a variety of epidermal processes, including epidermal lipid metabolism, the establishment of intercellular junctions and keratinocyte differentiation [3,4]. Patients with mutations in these genes develop symptoms, such as epidermal thickening, scaling, and increased susceptibility for concomitant inflammation of the skin [2,5].

Ichthyoses represent a large and heterogeneous group of hereditary DOC [6]. They are classified by inheritance patterns, their underlying pathomechanisms, and clinical features, and can be divided into syndromic and non-syndromic forms [7]. Ichthyosis vulgaris (IV) is the most common ichthyosis. It develops due to mutations in the filaggrin gene (*FLG, OMIM* #146700) and manifests with dry and scaly skin without overt signs of inflammation [8,9,10]. Histologically, IV epidermis is characterized by massive hyperkeratosis and a reduction or lack of keratohyalin granules [11]. In contrast to IV, autosomal recessive congenital ichthyoses (ARCI) are a group of rare skin disorders and include, amongst others, lamellar ichthyosis (LI) and harlequin ichthyosis (HI) [12]. Depending on the underlying mutation, manifesting symptoms are strongly variable [12]. Affected patients are characterized by patchy or generalized scaling that evolves into large scaly plaques and erythema. Netherton syndrome (NS), a syndromic form of ichthyosis, is caused by loss of function mutation in the serine protease inhibitor of Kazal type 5-gene (*SPINK5, OMIM* #256500) [13]. Functional loss of the SPINK5-encoded serine protease inhibitor lympho-epithelial Kazal type related inhibitor (LEKTI) leads to a compromised inhibition of kallikreins. The balance between protease activity and inhibition is shifted towards excessive proteolytic activity, leading to premature degradation of corneodesmosomes, impaired skin barrier integrity and skin inflammation [13].

The heterogeneous group of peeling skin syndromes (PSS) are autosomal recessive diseases that can be divided into localized and generalized forms [14]. Mutations of transglutaminase 5 (*TGM5, OMIM* #609796) and cystatin A (*CSTA, OMIM* #607936) have been identified in patients affected with localized PSS [15]. Within the generalized PSS, a further distinction can be made between a generalized non-inflammatory PSS (Type A PSS) and a generalized *PSS* with inflammation (Type *B PSS*) [15]. While Type *A PSS* is associated with loss-of-function mutations in the peptidase inhibitor clade B member 8 (*SERPINB8, OMIM* #617115) and filaggrin 2 (*FLG2, OMIM* #618084) gene, Type *B PSS* manifests after loss of function mutations in the corneodesmosin (*CDSN, OMIM* #270300) gene [16].

Hereditary palmoplantar keratodermas (PPKs) represent another heterogenic disease cluster [17]. The morphological characteristics of PPKs are reflected by hyperkeratosis and skin thickening on palms and soles [18]. Clinically, diffuse and circumscribed or punctiform keratosis can be distinguished. The spectrum of genes involved in the many different forms of PPKs is very broad [19]. Mutations of genes encoding the cornified envelope protein loricrin, several keratins, connexins, and other proteins have been identified in these skin disorders [17,19].

A common trait of most of these diseases is their early manifestation during infancy or young adulthood and the considerable physical and psychological burden for the affected patients. Therapeutic strategies mainly aim to improve the quality of life of the patients by relieving symptoms and by attempting to restore the functionality of the skin [20]. Approved treatment options include frequent use of emollients for moisturization, keratolytic compounds for the hyperkeratotic areas, and retinoids to promote normal keratinocyte differentiation [20]. In addition, prevention of bacterial and fungal skin infections is an important consideration when treating patients suffering from hereditary *DOC* [20]. As currently no cure for hereditary *DOCs* exists, validated disease models are highly desirable to extend the current understanding of the underlying pathomechanisms and to foster the development of novel and effective treatment options.

In this review, we provide an overview of different experimental models for the study of selected hereditary *DOC* and discuss their strengths and limitations. To focus on model systems, we have structured this review by available models and not by diseases. However, Table 1 also provides an overview of all models discussed in this review grouped by diseases.

## 2. Models to Study Hereditary DOC

**Figure 1:**
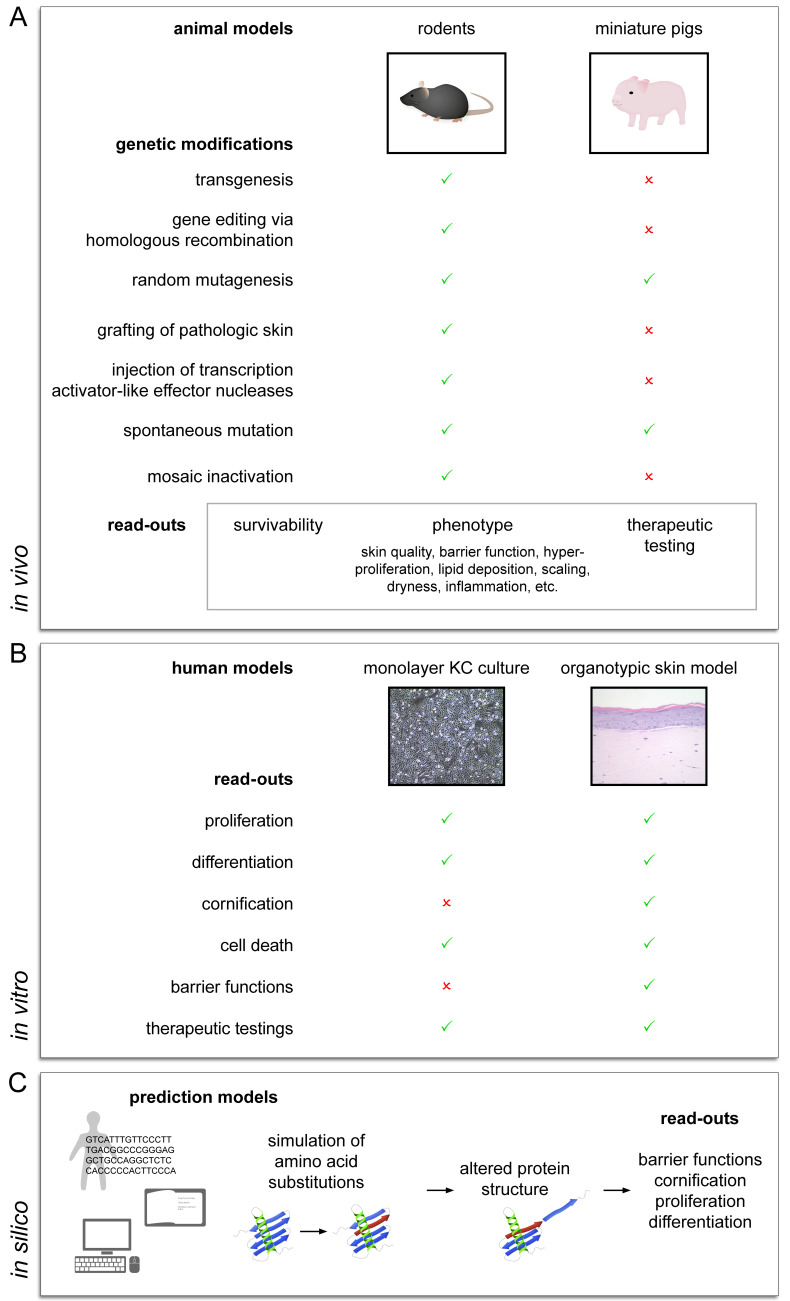
Schematic overview on experimental approaches for the study of hereditary disorders of cornification (**A**). Different genetic modifications are used to introduce cornification defects in animal-based models. Pathologic manifestations are assessed in terms of survivability, phenotypic changes, and response to therapy. (**B**) For in vitro models, human patient samples or primary human keratinocytes after target gene knockdowns are investigated in monolayer keratinocyte culture and three-dimensional organotypic skin models. (**C**) Computational methods are based on detection and characterization of sequence variants identified in patients and evaluation thereof in simulation and prediction programs.

Due to usually low patient numbers, studies on patients affected with hereditary *DOCs* are strongly limited and patient-derived samples are hardly available. Therefore, the establishment of valid disease models has been indispensable to study disease-related mechanisms and to develop new treatment options. Several experimental approaches have already been shown to significantly contribute to a better understanding of *DOCs*, including in vivo animal models, in vitro skin models, and in silico studies (Figure 1).

### 2.1. Animal Models

Animal models are most frequently used to study DOCs [20]. Generation of transgenic mice, gene editing through homologous recombination in mouse embryonic stem cells, and random mutagenesis with substances such as ethylnitrosourea (ENU) are used to introduce the respective mutations into the target genome [21]. Resulting phenotypic and functional changes, including survivability, structural changes of the epidermis, lipid composition, keratinocyte differentiation, presence of extracutaneous manifestations, and effectiveness of potential therapeutic strategies can be studied in these animals in detail. However, a considerable drawback of many in vivo mouse models of *DOCs* is the high neonatal mortality of mutated animals carrying such mutations [22]. Especially, diseases accompanied with compromised skin barrier function lead to early death mainly due to dehydration. [22,23] As an alternative to overcome the issue of early postnatal death, skin from newborn mice with early onset of lethal skin disorders can be grafted onto immunocompromised animals [24]. This approach allows for a longer and more profound study of the respective diseases and enables long term observations.

Although mouse models are widely used to study skin related pathologies, translation of basic and preclinical results from such experiments into clinics is, due to considerable anatomical and physiological differences between human and mouse skin, not always achievable [23,25]. Therefore, other animal models, such as porcine models, showing higher comparability to human skin, should be further developed. So far, such models are significantly more expensive and more difficult to handle. In addition, until now, these animal models are exclusively generated by random mutagenesis or spontaneous mutations [26].

#### 2.1.1. Ichthyosis Vulgaris

In 1999, Presland et al. described the flaky tail mouse that displays a spontaneous mutation in the Flg gene and shares several similarities with patients suffering from ichthyosis vulgaris [27]. Histopathological examination of skin samples from these mice showed large scales, attenuation of the epidermal granular layer, acanthosis, and hyperkeratosis [27]. Recently, Flg^−/−^ mice have been generated that only show dry, scaly skin with normal hydration of the stratum corneum and no increase in trans-epidermal water-loss (TEWL). Histological examination of these mice revealed shedding of the stratum corneum layers along with desquamation under mechanical stress. Additionally, the altered barrier function enhanced antigen penetration and sensitization. These features make the Flg^−/−^ mouse model not only valuable for IV research but even more so for studies on the inflammatory processes associated with atopic dermatitis [27].

#### 2.1.2. Autosomal Recessive Congenital Ichthyoses

Mutations in the transglutaminase 1 gene (TGM1) are the most frequently observed alterations in patients with ARCI [28]. Substitution of arginine with cysteine at codon 142 of TGM1 has previously been identified in a patient with lamellar ichthyosis. Using a transgenic mouse Nakamura and colleagues could reproduce this change in amino acid sequence and demonstrated severe impairments of stratum corneum barrier function and postnatal survival in homozygous mutant Tgm1^R142C/R142C^ mice [28]. Analysis of skin samples from these mice showed not only a profound decrease of transglutaminase 1 (TG1) protein levels but also diminished activity of the remaining protein in an in situ TG-activity assay [28]. Formation of the cornified envelope was completely abrogated and lipid lamellar structure was irregularly formed in the mutated animals, resulting in a barrier defect with increased *TEWL* [28]. Interestingly, the neonatal lethality in *Tgm1^R142C/R142C^* mice was comparable to previously studied *Tgm1^−/−^* mice [29]. This indicates that a structural change of the protein caused by an amino acid substitution mirror observed findings in *Tgm1*-deficient mice [29]. The *Tgm1*^−/−^ mouse model is also used to test the efficacy of therapeutic approaches such as enzyme replacement therapy and ex vivo gene therapy. Indeed, topical replacement therapy with recombinant human *TG1* (*rhTG1*) showed already promising results in a Tgm1-deficient humanized ARCI mouse model [30]. Treatment of these mice for 14 days showed considerable improvement of the ichthyosis phenotype and normalization of the ARCI skin. In addition to that, a restoration of endogenous TGM1 levels could also be detected in mutated keratinocytes from patients with *TGM1*-deficiency that had been treated with *rhTG1* [30].

Loss of function mutations of NIPA like domain containing 4 (Nipal4), a putative Mg^2+^ transporter, have been shown to be causative for lamellar ichthyosis [31]. The exact underlying mechanism, however, is still not completely understood. [31] In order to examine Nipal4-mediated ARCI, Honda and colleagues developed a transgenic mouse model of Nipal4-KO mice (Nipal4^−/−^), demonstrating a significant increase of TEWL in the knockout mice compared to Nipal4^+/+^ and Nipal4^+/−^ control animals [32]. Histological examinations identified ichthyosis-like features including increased numbers of stratum corneum layers and reduction of intercellular gaps due to impaired lipid lamella formation [32]. Since lipid composition and lipid lamellae are integral parts of proper skin barrier function, epidermal lipids were extracted and further analyzed with a particular focus on ceramides. Interestingly, the amount of acylceramides was significantly reduced in the epidermis of Nipal4^−/−^ mice [32]. Further analyses of lesional skin from patients with NIPAL4-ARCI also showed a decreased amount of acylceramide in the stratum spinosum and granulosum [33]. These data suggest that substitution or endogenous up-regulation of this lipid class might represent a new promising therapeutic approach.

Harlequin ichthyosis (HI) is the most severe form of ARCI and is caused by a mutation of the ATP-binding cassette *A12* gene (*ABCA12*) [34,35]. The ABCA12 protein is located within the lamellar granules in epidermal keratinocytes and mediates trafficking of lipids to the extracellular space, making it indispensable for epidermal barrier function. Use of Abca12-deficient mice is strongly limited by the early onset of post-natal death within a few hours after birth [35]. Interestingly, Wang et al. developed a porcine animal model which was more robust to the genetic defect [36]. They used the mutagenic substance ethylnitrosourea (ENU) to introduce a mutation in the *ABCA12* gene in Bama miniature pigs [36]. A disruption of lipid homeostasis was evident in the lipidomics analysis where levels of glucosylceramides, sphingosines, and free cholesterol were significantly increased in the skin of mutated pigs compared to controls [36]. Strikingly, oral administration of the synthetic retinoid acitretin strongly alleviated symptoms and reduced the severity of the HI, thereby extending the lifespan of these animals from 3 to up to 23 days. [36,37]. This model allows further characterization of the disease and enables testing of novel therapeutic approaches [37]. Thus, the gain in qualitative information generated by this model outperforms the increased efforts that come along with execution of large animal models [37].

#### 2.1.3. Autosomal Recessive Ichthyosis with Hypotrichosis

The transmembrane serine protease matriptase, encoded by the *ST14* gene (*OMIM* #602400), has been shown to be important for the proteolytic cascade processing pro-filaggrin to filaggrin. Mutation of the *ST14* gene leads to defects in epidermal barrier function and hair follicle development [38]. List and colleagues developed a matriptase-deficient mouse model, which showed classical ichthyosis-like traits such as dryness, wrinkles, and redness [39,40]. Functional assessment of outside-in barrier function revealed significantly higher toluidine blue dye penetration in genetically altered mice compared to control animals. In addition, an increased weight loss due to augmented TEWL could be registered in matriptase-deficient mice over time and resulted in their death within 48 h after birth due to compromised epidermal barrier [40]. To further study the mutation associated with epidermal phenotype, skin was transplanted from newborn matriptase knockout mice onto adult athymic mice [40]. They observed an ichthyosis-like phenotype in the recipients with thickening and compaction of the stratum corneum, severe acanthosis along with hyper- and parakeratosis, reflecting the phenotype of patients with a matriptase mutation [40].

#### 2.1.4. Syndromic Ichthyosis-Netherton Syndrome

Transgenic Spink5^−/−^ mice mimic hallmark features of human NS, including stratum corneum detachment due to desmosomal cleavage, profound skin barrier and hair shaft defects, and impaired epidermal keratinocyte differentiation [39,40]. Due to rapid postnatal lethality, this model does not allow to track phenotypic changes after a prolonged exposure to the environment. However, skin of newborn Spink5^−/−^ mice grafted onto nude mice and analyzed 5 weeks later retained an ichthyosis-like phenotype [41]. *KLK5* knock-out in these Spink5^−/−^ mice prevented lethality of newborn pups, restored skin barrier and epidermal structure and reversed skin inflammation, suggesting *KLK5* as a major therapeutic target for NS therapy [42]. In contrast to the study by Furio et al. [42], Kasparek et al. demonstrated that only the simultaneous ablation of the serine proteases *KLK5* and KLK7 completely inhibited barrier defects and postnatal lethality of Spink5-deficient mice [43].

Another interesting mouse model for NS is a Spink5-mosaic mouse model, generated by injection of transcription activator like effector nuclease targeting the Spink5 gene in mouse zygotes. Thereby the severity of the skin defect can be regulated by the amount of injected nucleases [44]. Spink5-mosaic mice mimic common symptoms of NS and, contrary to previously described Spink5-deficient mice, survive the neonatal period, which in turn allows the analysis of adult phenotypes including growth retardation, atopy, and hair defects.

Furthermore, the role of *KLK14* has been studied in a transgenic murine model, overexpressing human *KLK14* in the granular layer of epidermis. These mice presented major hair shaft defects together with aberrant keratinocyte differentiation and features of skin inflammation, underlying contribution of *KLK14* hyperactivity to clinical manifestations of NS [45].

#### 2.1.5. Peeling Skin Syndrome (PSS)

Animal models for PSS are scarce. Only CDSN^−/−^ mice have been reported to reflect most of the phenotypic findings observed in PSS. Corneodesmosin (CDSN) plays a crucial role in maintenance of cell-cell adhesion and corneodesmosome integrity in the upper epidermis and the stratum corneum. The impairment of skin barrier function, which arises from decreased mechanical resistance due to lack of cohesion between corneocytes, enables penetration of allergens and microbes and predisposes to atopic manifestations and skin infections [46]. CDSN^−/−^ mice display severe impairment of the barrier function and a low resistance to mechanical stress. The skin of neonatal pups in areas faced with high friction was very vulnerable for ruptures and tears, ultimately leading to early postnatal death. This is indicative of the intercellular fragility resulting from a loss of CDSN in the epidermis. Upon further histological examination, a complete detachment of the stratum corneum was observed in these areas [23,46].

#### 2.1.6. Palmoplantar Keratosis (PPK)

The epidermolytic palpmoplantar keratoderma (*EPPK,* OMIM #144200) is an inherited keratinopathy. It is the most common form of palmoplantar keratosis and accounts for approximately 50% of all cases. Genomic alterations in keratin 1 and keratin 9 are responsible for the disease, with the latter leading to a more severe phenotype. Patients suffering from *EPPK* show diffuse thickening of the skin on the palms and soles with distinct erythematous borders. Symptoms often develop early on after birth and are fully pronounced by the end of the second year at the latest. Lyu and colleagues introduced a small insertion-deletion (indel) mutant of KRT9 into mice [47]. A human EPPK-like phenotype was seen in *KRT9^+/mut^* and *KRT9^mut/mut^* mice with hyperpigmentation of the calluses and acanthosis in the hind-paws [47]. The paws of homozygous mutated mice showed expansion and thickening of the epidermis, massive hyperkeratosis and acanthosis [47]. This model served as a template for the development of mutant-specific short hairpin RNA (shRNA) which was able to relieve the EPPK-like phenotype effectively in *KRT9^+/mut^* mice [47].

KRT16-deficient mice are also often used for the study of non-epidermolytic palmoplantar keratosis (OMIM #613000). [48] PPK-like lesions, which include hyperkeratotic calluses on the front and hind paws, occur in these animals after 4–6 weeks [48]. The regions with high mechanical stress are particularly prone to callus formation leading to significant decrease in animal mobility [48]. Furthermore, a significant focal loss of epidermal barrier function was observed in the paws of KRT16^−/−^ mice [48].

In addition to keratins, a variety of other genes have been identified as drivers of PPKs. Loss of function of secreted Ly-6/uPAR-related protein 1 (SLURP1) is associated with a form of *PPK* known as mal de Meleda syndrome (*MDM*, OMIM #248300) [49]. Along with the typical patchy and dry skin on hands and soles, *MDM* is often accompanied by fungal infection, pronounced hyperhidrosis, and a maceration of hyperkeratotic masses with an unpleasant smell. Besides SLURP1-deficiency, Annan et al. could recently show that also SLURP2-deficient mice display histopathological features comparable to *PPK* [50]. Interestingly, inflammation and a higher susceptibility to local infection of the affected areas, which is a hallmark feature of MDM, were not observed in either of the models [50], leaving room for the assumption that infections are a consequence of the markedly thickened skin lesions rather than a direct result from deficiency in either of the SLURP genes.

### 2.2. In Vitro Models

**Figure 2:**
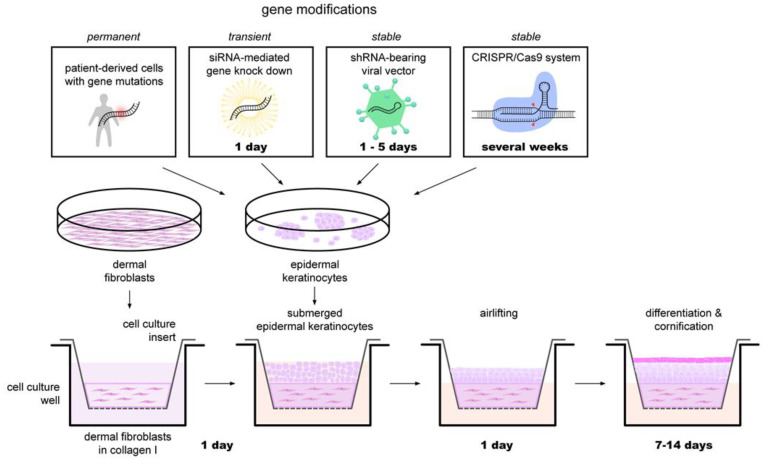
Three-dimensional organotypic skin models as a means for the study of hereditary disorders of cornification in vitro. Patient-derived cells or genetically modified primary human keratinocytes are the source for investigating hereditary cornification disorders with human organotypic skin models. Dermal fibroblasts are seeded in a cell culture insert submerged in a collagen I suspension. After two hours, the suspension is replaced with keratinocyte growth medium to equilibrate the collagen gel for another two hours. Next, keratinocytes are carefully layered onto the fibroblast gel matrix and cultivated overnight. Medium is then exchanged and added only to the outer cell culture well with the keratinocytes residing at the air–liquid interphase. Morphological and functional analysis of the three-dimensional organotypic skin models is done once differentiation and cornification have taken place.

Cell culture-based approaches such as keratinocyte monolayer cultures and three-dimensional organotypic skin models either seeded with keratinocytes isolated from patients [51] or genetically modified keratinocytes are important tools to study keratinocyte differentiation and epidermal barrier formation and function in vitro [51,52]. In this model, keratinocytes are grown on a collagen matrix, and fully differentiate during seven days of culture when exposed to air. An illustration of the composition and generation of skin models and knock-down skin models is depicted in Figure 2. Organotypic skin models mirror the three-dimensional stratified epithelium where keratinocytes normally reside and are therefore of particular importance when investigating physiological functions and pathological states of the epidermis, especially the epidermal barrier [52].

Combining an organotypic skin model with technologies such as small interfering RNA and CRISPR-Cas9 further extends the landscape of possible applications (Figure 2). Although in vivo use of these technologies had thus far posed rather difficult, the approaches in in vitro settings showed impressive results [53]. Implementation of these methods opened up new opportunities in the field of in vitro studies for *DOCs* as it enabled studies on the contribution of specific genes to keratinocyte differentiation and epidermal barrier formation. By using human keratinocytes, the reliance on animal models, along with their sometimes-questionable translatability of the obtained data, could be bypassed.

#### 2.2.1. Ichthyosis Models

Mildner et al. were the first to combine the technology of RNA interference for silencing epidermal genes in organotypic skin models to reproduce phenotypes previously observed in animal knockout models [54]. In their first study, they used siRNA to perform knock-down of the matriptase-1 gene in primary human keratinocytes and seeded them onto a collagen matrix for 7 days. The observed phenotype showed significantly increased thickening of the stratum corneum and impaired processing of filaggrin, thus offering a valuable alternative for the study of this skin disease [54,55].

One of the best studied genes using organotypic skin models is filaggrin [55]. The effects of filaggrin deficiency on the function of the epidermis have been investigated by several groups, using both, gene knock-downs, and cells from affected patients [56,57]. These studies have recently been summarized in a review article by Leman and coworkers [58].

Eckl et al. investigated different forms of autosomal recessive congenital ichthyosis (ARCI) in organotypic skin models by individual knock-downs of *TGM1* (OMIM #242300), arachidonate lipoxygenase 3 (*ALOXE3*, OMIM #606545), arachidonate 12-lipoxygenase, 12R Type (*ALOX12B*, OMIM #242100), NIPA like domain containing 4 (*NIPAL4*, OMIM #612281), ATP binding cassette subfamily A member 12 (*ABCA12*, OMIM #601277), and cytochrome P450 family 4 subfamily F member 22 (*CYP4F22*, OMIM #604777) in primary human keratinocytes [59]. Especially knock-down of *TGM1*, *ALOX12B*, and *ALOCE3* showed phenotypical alterations comparable to clinical features of *ARCI*, including thickening of the SC, earlier flattening of keratinocytes, and a more compact structure of the epidermis, suggesting them as useful in vitro models for ARCI. Youssef et al. investigated common gene expression changes in two different rat epidermal keratinocyte ARCI models [60]. They compared two different Arachidonate 12-lipoxygenase (*Alox12b*) short hairpin RNA (shRNA) knock-down rat epidermal keratinocytes cell lines to a *TGM1-shRNA* knockdown line in organotypic skin models [60]. Both approaches showed phenotypic similarities and analysis of differentially expressed genes revealed 78 commonly upregulated genes. Mdm2, which had previously been shown to cause hyperkeratosis in *MdM2*-overexpressing mice, was amongst these genes [61] and inhibition of *Mdm2* by nutlin-3 prevented hyperkeratosis in these rat *ARCI* models [60]. Thus, using a specific knock-down skin model identified a novel treatment option to target hyperkeratosis in *ARCI* and other hyperkeratotic disorders.

Recently, Mohamad et al. demonstrated that patients with loss-of-function mutations of the serpin family A member 12 (*SERPINA12*) gene developed palmoplantar keratoderma [62]. Knock-down of *SERPINA12* in skin models induced acanthosis and hyperkeratosis, making it a valuable model for functional studies of palmoplantar keratoderma in an in vitro model [62].

#### 2.2.2. Peeling Skin Syndrome

Studies on PSS in skin models are difficult since knock-down of genes associated with PSS might not lead to the expected morphological alterations. Although alterations in gene expression patterns and structural cellular components can be observed, the typical dissociation of epidermal layers might not be present without additional mechanical stress [23]. A recent publication by Mohamad showed this phenomenon for *FLG2* and CDSN-deficient skin models [63]. These models represent important tools to study disease-associated mechanisms in human cells.

#### 2.2.3. The Use of Skin Models to Identify the Role of So Far Uncharacterized Genes for *DOC*

Skin models allow rapid testing of the contribution of specific molecules to epidermal development and barrier function. Compared to animal experiments, this experimental setup can be used for screening of several genes of interest within a short time frame. Therefore, this method is being increasingly used to study the role of poorly investigated molecules on epidermal function.

Recently, we found that knock-down of cornifelin (*CNFN*) led to a phenotype comparable to those observed in *PSS* [64]. The skin models showed reduced *CDSN* expression and structural alterations in corneodesmosomes, leading to enhanced tissue dissociation upon mechanical stress. In addition, we could show that *CNFN* plays an important role for the epithelial integrity of oral mucosa [64]. While *CNFN* had previously been solely associated with keratinocyte differentiation our study, using organotypic knock-down skin models, identified a putative role of *CNFN* for *PSS* [64].

In another recent study we identified the whey acidic protein WFDC12 as a novel epidermal serine protease inhibitor, which might be involved in the pathogenesis of NS [65].

### 2.3. In Silico Odels

In silico studies using sequence variant predictions programs such as Modeller [66], SIFT [67], PolyPhen-2 [68], and MutationTaster [69] have been shown to be useful tools to determine the effects of amino acid substitutions on protein structure. Although such studies on *DOCs* are still scarce, we found one study evaluating the pathogenicity of mutational changes in genes responsible for hereditary cornification disorders. In the study of Borska et al., DNA sequencing of 59 patients, clinically diagnosed with an inherited ichthyosis, were used to analyze their expected protein structures and associate them to disease severity. [70] Strikingly, their in silico structure analysis revealed that proteins with significant three-dimensional structural alterations correlated to 97.4% with pathogenic sequence variants, and that mild alterations of protein structures correlated to 100% with benign sequence variants [70].

Therefore, detailed in silico structural analysis of proteins, based on DNA-sequencing of patient material might be a useful tool to predict disease severity and treatment recommendations.

## 3. Conclusions

Hereditary *DOCs* bear a great burden to affected individuals, and due to their low frequency, studies in humans are scarce. Therefore, valid experimental models are needed to better understand the underlying pathomechanisms and develop new therapeutic strategies for patients. In vivo animal approaches offer valuable insights into the complex interactions of the skin with other organ systems, however, there are considerable limitations to their use for the study of hereditary *DOCs*. Especially in settings involving small animal models, the early onset of postnatal death makes the study of long-term complications difficult. Additionally, interspecies differences in structural composition and physiological function of the epidermis further complicate the translation of findings of experimental in vivo models to clinical settings. Following the European ban on animal testing, the 3R principles (Replacement, Reduction, Refinement) for research involving animal models were implemented. Ever since, non-animal-based models have moved to the frontline and gradually replaced animal-based approaches. Three-dimensional human organotypic skin models are well suited to investigate biological processes involved in hereditary *DOCs*. By using gene silencing methods, several of the described pathologies have already been reproduced in human organotypic skin models. The use of human material represents a major advantage of this method. Another big advantage is its potential to easily and rapidly screen for potential candidate genes that so far may not have been associated with hereditary *DOCs*. A disadvantage, however, is that interactions with or the influence of other cell types or organs cannot be studied in these in vitro skin models. The use of in silico methods to study *DOCs* is still at the beginning. However, it is very likely that new bioinformatics tools and in silico predictions will strongly impact the research on *DOCs* in the near future.

Although the establishment of models for *DOCs* remains challenging, constant progress in developing new methods and new models has led to a better understanding of these pathologies.

## Figures and Tables

**Table 1:** Summary of the presented experimental models.

Pathology	Gene	Phenotypic Characteristics	Phenotype Observed in Respective Model			
			ft/ft Mouse	Flg KO Mouse	FLG Knockdown Organotypic Skin model	
**Ichthyosis vulgaris**	*FLG*	Dry and scaly skin	✓	✓	✗	
		Skin barrier defects	✓	✓	✓	
		Thick stratum corneum	✓	✓	✓	
		Impaired formation of lamellar bodies	✓	✓	✓	
		Loss of keratohyalin granules	✓	✓	✓	
			Tgm1^R142C/R142C^ Mouse	Tgm KO mouse	Nipal4 KO mouse	Gene Knockdown Organotypic Skin Model
**Lamellar Ichthyosis**	*TGM1* *NIPAL4* *ALOXE3* *ALOX12B*	Disturbed epidermal differentiation	✓	✓	✓	✓
		Stratum corneum thickening	✓	✓	✓	✓
		Dysfunctional epidermal skin barrier	✓	✓	✓	✓
		Altered lipid composition	✓	✓	✓	✗
			Bama Miniature Pig			
**Harlequin Ichthyosis**	*ABCA12*	Sclerotic and dry skin	✓			
		Hyperkeratotic stratum corneum	✓			
		Increased TEWL	✓			
		Impaired barrier function	✓			
			ST14 KO Mouse	ST14-Knockdown Organotypic Skin Model		
**ARCI with hypotrichosis (ARIH)**	*ST14*	Increased skin thickness	✓	✓		
		Disrupted terminal differentiation	✓	✓		
		Hair shaft defect	✓	✓		
			Spink5 KO Mouse	Spink5-Mosaic Mouse		
**Netherton Syndrom**	*SPINK5*	Atopy	✗	✓		
		Skin barrier impairment	✓	✓		
		Hair shaft defects	✗	✓		
		Early postnatal death	✓	✗		
			CDSN KO Mouse	FLG2 Organotypic Skin Model		
**Type-B Peeling skin syndrome**	*CDSN* *FLG2*	Barrier function impairment	✓	✓		
		Decreased mechanical resistance	✓	✓		
		Detachment of stratum corneum	✓	✓		
		Postnatal death	✓	✗		
			KRT9^mut/mut^ Mouse	KRT16 KO Mouse		
**Epidermiolytic palmoplantar keratoderma**	*KRT1* *KRT9* *KRT16*	Hind-paw acanthosis	✓	✓		
		Hyperkeratosis	✓	✓		
		Callus formation	✓	✓		
		Hyperpigmentation	✓	✓		
		Epidermal thickening	✓	✓		
			SERPINA12-Knockdown Organotypic Skin Model			
**Autosomal recessive palmoplantar keratoderma**	*SERPINA12*	Acanthosis	✓			
		Hyperkeratosis	✓			
		Dysfunctional differentiation	✓			
			SLURP1 KO Mouse	SLURP2 KO Mouse	SLURP1/SLURP2 Double-KO Mouse	
**Mal de Meleda**	*SLURP1* *SLURP2*	Paw hyperkeratosis	✓	✓	✓	
		Stratum granulosum decarmation	✓	✓	✓	
		Inflammation	✗	✗	✗	

## References

[1] Ammirati C.T., Mallory S.B. (1998). The major inherited disorders of cornification: New advances in pathogenesis. Dermatol. Clin..

[2] Mathes E.F., Spring S., Friedland R., Teng J., Marqueling A., Benjamin L. (2017). PAS. Hereditary Disorders of Cornification. Therapy in Pediatric Dermatology.

[3] Van Smeden J., Janssens M., Gooris G.S., Bouwstra J.A. (2014). The important role of stratum corneum lipids for the cutaneous barrier function. Biochim. Biophys. Acta–Mol. Cell Biol. Lipids.

[4] Candi E., Schmidt R., Melino G. (2005). The cornified envelope: A model of cell death in the skin. Nat. Rev. Mol. Cell Biol..

[5] Bouwstra J.A., Ponec M. (2006). The skin barrier in healthy and diseased state. Biochim. Biophys. Acta-Biomembr..

[6] Schmuth M., Martinz V., Janecke A.R., Fauth C., Schossig A., Zschocke J., Gruber R. (2013). Inherited ichthyoses/generalized Mendelian disorders of cornification. Eur. J. Hum. Genet..

[7] Oji V., Tadini G., Akiyama M., Blanchet Bardon C., Bodemer C., Bourrat E., Coudiere P., DiGiovanna J., Elias P., Fischer J. (2010). Revised nomenclature and classification of inherited ichthyoses: Results of the First Ichthyosis Consensus Conference in Sorze 2009. J. Am. Acad. Dermatol..

[8] Takeichi T., Akiyama M. (2016). Inherited ichthyosis: Non-syndromic forms. J. Dermatol..

[9] McLean W.H.I. (2016). Filaggrin failure—From ichthyosis vulgaris to atopic eczema and beyond. Br. J. Dermatol..

[10] Thyssen J.P., Godoy-Gijon E., Elias P.M. (2013). Ichthyosis vulgaris: The filaggrin mutation disease. Br. J. Dermatol..

[11] Rodríguez-Pazos L., Ginarte M., Vega A., Toribio J. (2013). Autosomal recessive congenital ichthyosis. Actas Dermosifiliogr..

[12] Hovnanian A. (2013). Netherton syndrome: Skin inflammation and allergy by loss of protease inhibition. Cell Tissue Res..

[13] Oji V., Metze D., Traupe H., Griffiths C.E.M., Barker J., Bleiker T., Chalmers R., Creamer D. (2016). Inherited Disorders of Cornification. Rook’s Textbook of Dermatology.

[14] Has C. (2018). Peeling Skin Disorders: A Paradigm for Skin Desquamation. J. Investig. Dermatol..

[15] Samuelov L., Sprecher E. (2014). Peeling off the genetics of atopic dermatitis-like congenital disorders. J. Allergy Clin. Immunol..

[16] Has C., Technau-Hafsi K. (2016). Palmoplantar keratodermas: Clinical and genetic aspects. J. Der Dtsch. Dermatologischen Gesellschaft.

[17] Thomas L.J., Freeman A., O’Toole E.A., McGrath J.A., Perrett C.M. (2018). Inherited palmoplantar keratodermas: The heart of the matter. Clin. Exp. Dermatol..

[18] Sakiyama T., Kubo A. (2016). Hereditary palmoplantar keratoderma “clinical and genetic differential diagnosis”. J. Dermatol..

[19] Oji V., Preil M.L., Kleinow B., Wehr G., Fischer J., Hennies H.C., Hausser I., Breitkreuz D., Aufenvenne K., Stieler K. (2017). S1 guidelines for the diagnosis and treatment of ichthyoses—Update. J. Ger. Soc. Dermatol..

[20] Avci P., Sadasivam M., Gupta A., De Melo W.C., Huang Y.Y., Yin R., Chandran R., Kumar R., Otufowora A., Nyame T. (2013). Animal models of skin disease for drug discovery. Expert Opin. Drug Discov..

[21] Chen J., Roop D.R. (2008). Genetically engineered mouse models for skin research: Taking the next step. J. Dermatol. Sci..

[22] Hewett D.R., Simons A.L., Mangan N.E., Jolin H.E., Green S.M., Fallon P.G., McKenzie A.N.J. (2005). Lethal, neonatal ichthyosis with increased proteolytic processing of filaggrin in a mouse model of Netherton syndrome. Hum. Mol. Genet..

[23] Leclerc E.A., Huchenq A., Mattiuzzo N.R., Metzger D., Chambon P., Ghyselinck N.B., Serre G., Jonca N., Guerrin M. (2009). Corneodesmosin gene ablation induces lethal skinbarrier disruption and hair-follicle degeneration related to desmosome dysfunction. J. Cell Sci..

[24] List K., Szabo R., Wertz P.W., Segre J., Haudenschild C.C., Kim S.Y., Bugge T.H. (2003). Loss of proteolytically processed filaggrin caused by epidermal deletion of Matriptase/MT-SP1. J. Cell Biol..

[25] Schneider M.R. (2012). Genetic mouse models for skin research: Strategies and resources. Genesis.

[26] Hai T., Cao C., Shang H., Guo W., Mu Y., Yang S., Zhang Y., Zheng Q., Zhang T., Wang X. (2017). Pilot study of large-scale production of mutant pigs by ENU mutagenesis. eLife.

[27] Presland R.B., Boggess D., Lewis S.P., Hull C., Fleckman P., Sundberg J.P. (2000). Loss of normal profilaggrin and filaggrin in flaky tail (ft/ft) mice: An animal model for the filaggrin-deficient skin disease ichthyosis vulgaris. J. Investig. Dermatol..

[28] Nakagawa N., Yamamoto M., Imai Y., Sakaguchi Y., Takizawa T., Ohta N., Yagi N., Hatta I., Takizawa T., Takeda J. (2012). Knocking-in the R142C mutation in transglutaminase 1 disrupts the stratum corneum barrier and postnatal survival of mice. J. Dermatol. Sci..

[29] Matsuki M., Yamashita F., Ishida-Yamamoto A., Yamada K., Kinoshita C., Fushiki S., Ueda E., Morishima Y., Tabata K., Yasuno H. (1998). Defective stratum corneum and early neonatal death in mice lacking the gene for transglutaminase 1 (keratinocyte transglutaminase). Proc. Natl. Acad. Sci. USA.

[30] Aufenvenne K., Larcher F., Hausser I., Duarte B., Oji V., Nikolenko H., Del Rio M., Dathe M., Traupe H. (2013). Topical enzyme-replacement therapy restores transglutaminase 1 activity and corrects architecture of transglutaminase-1-deficient skin grafts. Am. J. Hum. Genet..

[31] Dahlqvist J., Westermark G.T., Vahlquist A., Dahl N. (2012). Ichthyin/NIPAL4 localizes to keratins and desmosomes in epidermis and Ichthyin mutations affect epidermal lipid metabolism. Arch. Dermatol. Res..

[32] Honda Y., Kitamura T., Naganuma T., Abe T., Ohno Y., Sassa T., Kihara A. (2018). Decreased Skin Barrier Lipid Acylceramide and Differentiation-Dependent Gene Expression in Ichthyosis Gene Nipal4-Knockout Mice. J. Investig. Dermatol..

[33] Murase Y., Takeichi T., Kawamoto A., Tanahashi K., Okuno Y., Takama H., Shimizu E., Ishikawa J., Ogi T., Akiyam M. (2020). Reduced stratum corneum acylceramides in autosomal recessive congenital ichthyosis with a NIPAL4 mutation. J. Dermatol. Sci..

[34] Zhang L., Ferreyros M., Feng W., Hupe M., Crumrine D.A., Chen J., Elias P.M., Holleran W.M., Niswander L., Hohl D. (2016). Defects in stratum corneum desquamation are the predominant effect of impaired ABCA12 function in a novel mouse model of Harlequin Ichthyosis. PLoS ONE.

[35] Smyth I., Hacking D.F., Hilton A.A., Mukhamedova N., Meikle P.J., Ellis S., Satterley K., Collinge J.E., de Graaf C.A., Bahlo M. (2008). A mouse model of harlequin ichthyosis delineates a key role for Abca12 in lipid homeostasis. PLoS Genet.

[36] Wang X., Cao C., Li Y., Hai T., Jia Q., Zhang Y., Zheng Q., Yao J., Qin G., Zhang Y. (2019). A harlequin ichthyosis pig model with a novel ABCA12 mutation can be rescued by acitretin treatment. J. Mol. Cell Biol..

[37] Lee K. (2019). A novel pig model capturing clinical symptoms of harlequin ichthyosis. J. Mol. Cell Biol..

[38] Basel-Vanagaite L., Attia R., Ishida-Yamamoto A., Rainshtein L., Ben Amitai D., Lurie R., Pasmanik-Chor M., Indelman M., Zvulunov A., Saban S. (2007). Autosomal recessive ichthyosis with hypotrichosis caused by a mutation in ST14, encoding type II transmembrane serine protease matriptase. Am. J. Hum. Genet.

[39] List K., Currie B., Scharschmidt T.C., Szabo R., Shireman J., Molinolo A., Cravatt B.F., Segre J., Bugge T.H. (2007). Autosomal ichthyosis with hypotrichosis syndrome displays low matriptase proteolytic activity and is phenocopied in ST14 hypomorphic mice. J. Biol. Chem..

[40] List K., Haudenschild C.C., Szabo R., Chen W.J., Wahl S.M., Swaim W., Engelholm L.H., Behrendt N., Bugge T.H. (2002). Matriptase/MT-SP1 is required for postnatal survival, epidermal barrier function, hair follicle development, and thymic homeostasis. Oncogene.

[41] Descargues P., Draison C., Bonnart C., Kreft M., Kishibe M., Ishida-Yamamoto A., Elias P., Barrandon Y., Zambruno G., Sonnenberg A. (2005). Spink5-deficient mice mimic Netherton syndrome through degradation of desmoglein 1 by epidermal protease hyperactivity. Nat. Genet..

[42] Furio L., Pampalakis G., Michael I.P., Nagy A., Sotiropoulou G., Hovnanian A. (2015). KLK5 Inactivation Reverses Cutaneous Hallmarks of Netherton Syndrome. PLoS Genet.

[43] Kasparek P., Ileninova Z., Zbodakova O., Kanchev I., Benada O., Chalupsky K., Brattsand M., Beck I.M., Sedlacek R. (2017). KLK5 and KLK7 Ablation Fully Rescues Lethality of Netherton Syndrome-Like Phenotype. PLoS Genet.

[44] Kasparek P., Ileninova Z., Haneckova R., Kanchev I., Jenickova I., Sedlacek R. (2016). A viable mouse model for Netherton syndrome based on mosaic inactivation of the Spink5 gene. Biol. Chem..

[45] Gouin O., Barbieux C., Leturcq F., Bonnet des Claustres M., Petrova E., Hovnanian A. (2020). Transgenic Kallikrein 14 Mice Display Major Hair Shaft Defects Associated with Desmoglein 3 and 4 Degradation, Abnormal Epidermal Differentiation, and IL-36 Signature. J. Investig. Dermatol..

[46] Zaafouri S., Pichery M., Huchenq A., Valentin F., Oji V., Mazereeuw-Hautier J., Serre G., Jonca N. (2018). Transcriptomic Analysis of Two Cdsn-Deficient Mice Shows Gene Signatures Biologically Relevant for Peeling Skin Disease. J. Investig. Dermatol..

[47] Lyu Y.S., Shi P.L., Chen X.L., Tang Y.X., Wang Y.F., Liu R.R., Luan X.R., Fang Y., Mei R.H., Du Z.F. (2016). A Small Indel Mutant Mouse Model of Epidermolytic Palmoplantar Keratoderma and Its Application to Mutant-specific shRNA Therapy. Mol. Ther. —Nucleic Acids.

[48] Lessard J.C., Coulombe P.A. (2012). Keratin 16-null mice develop palmoplantar keratoderma, a hallmark feature of pachyonychia congenita and related disorders. J. Investig. Dermatol..

[49] Fischer J. (2001). Mutations in the gene encoding SLURP-1 in Mal de Meleda. Hum. Mol. Genet..

[50] Allan C.M., Heizer P.J., Jung C.J., Tu Y., Tran D., Young L.C., Fong L.G., de Jong P.J., Beigneux A.P., Young S.G. (2018). Palmoplantar keratoderma in Slurp1/Slurp2 double-knockout mice. J. Dermatol. Sci..

[51] Niehues H., Bouwstra J.A., El Ghalbzouri A., Brandner J.M., Zeeuwen P.L.J.M., van den Bogaard E.H. (2018). 3D skin models for 3R research: The potential of 3D reconstructed skin models to study skin barrier function. Exp. Dermatol..

[52] Klicks J., von Molitor E., Ertongur-Fauth T., Rudolf R., Hafner M. (2017). In vitro skin three-dimensional models and their applications. J. Cell. Biotechnol..

[53] Ain Q.U., Campos E.V.R., Huynh A., Witzigmann D., Hedtrich S. (2020). Gene Delivery to the Skin—How Far Have We Come?. Trends Biotechnol..

[54] Mildner M., Ballaun C., Stichenwirth M., Bauer R., Gmeiner R., Buchberger M., Mlitz V., Tschachler E. (2006). Gene silencing in a human organotypic skin model. Biochem. Biophys. Res. Commun..

[55] Pendaries V., Malaisse J., Pellerin L., Le Lamer M., Nachat R., Kezic S., Schmitt A.M., Paul C., Poumay Y., Serre G. (2014). Knockdown of filaggrin in a three-dimensional reconstructed human epidermis impairs keratinocyte differentiation. J. Investig. Dermatol..

[56] Vávrová K., Henkes D., Strüver K., Sochorová M., Školová B., Witting M.Y., Friess W., Schreml S., Meier R.J., Schäfer-Korting M. (2014). Filaggrin deficiency leads to impaired lipid profile and altered acidification pathways in a 3D skin construct. J. Investig. Dermatol..

[57] Stefan Blunder Ralph Rühl S.D. (2017). Alterations in Epidermal Eicosanoid Metabolism Contribute to Inflammation and Impaired Late Differentiation in FLG-Mutated Atopic Dermatitis Stefan. J. Investig. Dermatol..

[58] Leman G., Moosbrugger-Martinz V., Blunder S., Pavel P., Dubrac S. (2019). 3D-Organotypic Cultures to Unravel Molecular and Cellular Abnormalities in Atopic Dermatitis and Ichthyosis Vulgaris. Cells.

[59] Eckl K.M., Alef T., Torres S., Hennies H.C. (2011). Full-thickness human skin models for congenital ichthyosis and related keratinization disorders. J. Investig. Dermatol..

[60] Youssef G., Ono M., Brown S.J., Kinsler V.A., Sebire N.J., Harper J.I., O’Shaughnessy R.F.L. (2014). Identifying a hyperkeratosis signature in autosomal recessive congenital Ichthyosis: Mdm2 inhibition prevents hyperkeratosis in a rat ARCI model. J. Investig. Dermatol..

[61] Alkhalaf M., Ganguli G., Messaddeq N., Le Meur M., Wasylyk B. (1999). MDM2 overexpression generates a skin phenotype in both wild type and p53 null mice. Oncogene.

[62] Mohamad J., Sarig O., Malki L., Rabinowitz T., Assaf S., Malovitski K., Shkury E., Mayer T., Vodo D., Peled A. (2020). Loss-of-Function Variants in SERPINA12 Underlie Autosomal Recessive Palmoplantar Keratoderma. J. Investig. Dermatol..

[63] Mohamad J., Sarig O., Godsel L.M., Peled A., Malchin N., Bochner R., Vodo D., Rabinowitz T., Pavlovsky M., Taiber S. (2018). Filaggrin 2 Deficiency Results in Abnormal Cell-Cell Adhesion in the Cornified Cell Layers and Causes Peeling Skin Syndrome Type, A.J. Investig. Dermatol..

[64] Wagner T., Beer L., Gschwandtner M., Eckhart L., Kalinina P., Laggner M., Ellinger A., Gruber R., Kuchler U., Golabi B. (2019). The Differentiation-Associated Keratinocyte Protein Cornifelin Contributes to Cell-Cell Adhesion of Epidermal and Mucosal Keratinocytes. J. Investig. Dermatol..

[65] Kalinina P., Vorstandlechner V., Buchberger M., Eckhart L., Lengauer B., Golabi B., Laggner M., Hiess M., Sterniczky B., Födinger D. (2020). The whey acidic protein WFDC12 is specifically expressed in terminally differentiated keratinocytes and regulates epidermal serine-protease activity. J. Investig. Dermatol..

[66] Webb B., Sali A. (2016). Comparative protein structure modeling using MODELLER. Curr. Protoc. Bioinforma.

[67] Sim N.L., Kumar P., Hu J., Henikoff S., Schneider G., Ng P.C. (2012). SIFT web server: Predicting effects of amino acid substitutions on proteins. Nucleic Acids Res..

[68] Adzhubei I., Jordan D.M., Sunyaev S.R. (2013). Predicting functional effect of human missense mutations using PolyPhen-2. Curr. Protoc. Hum. Genet..

[69] Schwarz J.M., Rödelsperger C., Schuelke M., Seelow D. (2010). MutationTaster evaluates disease- causing potential of sequence alterations. Nat. Methods.

[70] Borská R., Pinková B., Réblová K., Bučková H., Kopečková L., Němečková J., Puchmajerová A., Malíková M., Hermanová M., Fajkusová L. (2019). Inherited ichthyoses: Molecular causes of the disease in Czech patients. Orphanet. J. Rare Dis..

